# Medical students’ perceptions of a novel institutional incident reporting system: A thematic analysis

**DOI:** 10.1007/s40037-017-0369-6

**Published:** 2017-08-16

**Authors:** Morris Gordon, Dillan Parakh

**Affiliations:** 1 Families Division, Blackpool Teaching Hospitals NHS Foundation Trust, Blackpool, UK; 2 School of Medicine, University of Central Lancashire, Preston, UK; 3 School of Psychology, University of Central Lancashire, Preston, UK

**Keywords:** Patient safety, Incident reporting, Professionalism, Human factors, Non-technical skills

## Abstract

**Background:**

Errors in healthcare are a major patient safety issue, with incident reporting a key solution. The incident reporting system has been integrated within a new medical curriculum, encouraging medical students to take part in this key safety process. The aim of this study was to describe the system and assess how students perceived the reporting system with regards to its role in enhancing safety.

**Methods:**

Employing a thematic analysis, this study used interviews with medical students at the end of the first year. Thematic indices were developed according to the information emerging from the data. Through open, axial and then selective stages of coding, an understanding of how the system was perceived was established.

**Results:**

Analysis of the interview specified five core themes: (1) Aims of the incident reporting system; (2) internalized cognition of the system; (3) the impact of the reporting system; (4) threshold for reporting; (5) feedback on the systems operation. Selective analysis revealed three overriding findings: lack of error awareness and error wisdom as underpinned by key theoretical constructs, student support of the principle of safety, and perceptions of a blame culture.

**Conclusions:**

Students did not interpret reporting as a manner to support institutional learning and safety, rather many perceived it as a tool for a blame culture. The impact reporting had on students was unexpected and may give insight into how other undergraduates and early graduates interpret such a system. Future studies should aim to produce interventions that can support a reporting culture.

## What this paper adds

This paper describes the implementation of an incident reporting system in medical school that is described in a manner that supports dissemination. Student perceptions are that such a system can enhance safety in general, but they paradoxically perceived a blame culture and did not discuss learning from error. Changes have been made to the system and future work must examine how to train students for their roles in incident reporting within healthcare to enhance safety, and whether this system can be augmented with other educational methods to support students in gaining such competence.

## Introduction

Recent research has illuminated the scale of errors in healthcare, with 1.5 million medical errors reported in the US each year [1] and 400,000 errors occurring in the UK, resulting in 35,000 deaths [2]. This has been estimated to incur a financial burden in the UK of £ 2 billion per annum [3], with the intangible cost to patients and their families much greater [4]. These findings suggest the failure of current strategies in reducing error, thus jeopardizing patient safety. The challenge of how this can be addressed remains to be answered [5].

Traditionally, patient safety initiatives have focussed on individuals making mistakes who are ‘blamed, named and retrained’. The majority of errors are overlooked or unreported [6] and therefore deviant behaviours become reinforced in non-blamed individuals [7]. This creates a culture of both lack of understanding of how errors happen and fear of reporting, paradoxically reducing the number of incidents reported [8]. In addressing these issues, the psychological discipline of human factors ergonomics has emerged [9]. Non-technical skills are a subset of human factors that focus on individuals, and aim to promote safety by teaching social and cognitive skills to healthcare workers so they can recognize and prevent potential errors with a ‘human factors’ understanding of health [10]. This requires detailed and accurate reporting of actual and potential incidents. Such reporting facilities an awareness of errors amongst staff and informs institutional strategies for human factors, a key element to facilitate enhanced non-technical skills [11]. The elements that lead to learning non-technical skills have been combined to create the SECTORS model [5], which describes key content areas, pedagogical methods and outcomes for such learning.

The World Health Organization (WHO) patient safety curriculum [12] states that teaching students how to report errors is central to recognizing and minimizing errors through human factors. According to the General Medical Council (GMC) Tomorrow’s Doctors [13], graduating UK medical students should be able to: ‘Promote, monitor and maintain health and safety in the clinical setting, understanding how errors can happen in practice, applying the principles of quality assurance, clinical governance and risk management to medical practice, and understanding responsibilities within the current systems for raising concerns about the safety and quality’. Furthermore, Health Education England [14] states that incident reporting is a pillar of patient safety, recommending ‘consolidation of both professionalism and human factors across training and education’ and ‘having the ability to raise concerns’. Mitchell et al. [15] recently highlighted the effectiveness of incident reporting in promoting safety in practice, both by identifying patterns of infrequent serious events and understanding causes of error. Despite this key focus on reporting, there is a paucity of research reporting education on such systems [16]. There has been one report in Dentistry [17], although this was focussed on identifying unprofessional behaviour by staff rather than students.

At the University of Central Lancashire School of Medicine, a patient safety curriculum was produced for the MBBS program in 2015, based on GMC and WHO outcomes. Rather than simply delivering conceptual teaching about incident reporting, a decision was made to implement an incident reporting system within the school that students also used. This was started 6 months prior to the first intake of students and has now been running for 2 years. The structured event reporting form (SERF) system is an online open access portal which staff, students and the public use to report any significant events. The three broad categories are: positive feedback, safety and wellbeing or professionalism alerts (which is for staff only and forms a low level professionalism lapse reporting system). Students discuss such reports with personal academic advisors or, if confidential, with a pastoral tutor from the school. In addition to learning about concepts and understanding the human factors science behind incident reporting, students participate in the process themselves and reports can be made about central faculty, a wider body of clinical tutors or events impacting anyone involved with the school. Reports are triaged at Level 1 (action plan within 2 weeks), Level 2 (action plan within 1 week) and Level 3 (action plan within 24 h). This triage judgement does not link to outcomes or predetermine an action, but merely guides the timeline and faculty members who are informed about such reports. Table 1 gives overall data for the first 6 months of operation and some examples. An audit of these data was completed by two faculty members not involved with the reporting system. They agreed with all but one of the assigned triage levels, which was a safeguarding concern that was downgraded. Since its introduction, reporting frequency initially increased exponentially and has now stabilized at five reports through the SERF per week.

**Table 1:** Reports and examples in the first 6 months of operation

Level	Positive feedback	Professionalism alert	Safeguarding/welfare
Level 1	28 Support and assistance to the delivery team (preparation of classrooms); sharing of sensitive information in a professional and appropriate manner; to assist fellow students’ learning; a student report in respect of her first placement supervisor	15 Including disruption in class, late arrival; failure to engage in small group learning activities	2 Student comfort with handwashing on placement
Level 2		1 Failure to demonstrate appropriate behaviour/sensitivity during a classroom presentation	6 Students witnessing a failed resuscitation within a first clinical visit; student concerns around stress levels/ability to cope; family-related issues
Level 3		N/A	1 A student health concern

This system moves past existing systems that aim to teach students that reporting is not about blaming, but about institutional learning and safeguarding themselves, colleagues and patients and sharing information as key to achieving this. This is underpinned by theory in the field [11] and builds on recent postgraduate examples [18]. Thus, they can acquire personal non-technical skills in ‘error awareness’, ‘error wisdom’ and combat identified theoretical constructs that lead to error [19]. The aim of this study was to assess, after one year of operation, how students perceived the reporting system, particularly with respect to safety.

## Methods

### Design

The whole first year cohort of 35 medical students of the MBBS program at the University of Central Lancashire medical school were invited to interviews. They had all consented previously to periodic invitations for such interviews as part of a wider study approved by the university ethics board. A qualitative methodology employing thematic analysis for the research was used, situated within a relativist research paradigm [20].

The focus group interviews, which each lasted approximately 10 min, were recorded with an audio recorder. All interviews were completed by the first author within a dedicated interview room and were recorded digitally. After completion, all interviews were transcribed, leading to eight transcripts. The interviews focused on the reporting system. The transcribing of the interviews removed all identifiers with pseudo-anonymous participant numbers used with no way to identify the original participants. Following transcription and removal of any identifiers, the original audio recordings were destroyed and only the pseudo-anonymized transcripts retained. Following collection and processing, the data were coded using Nvivo software (QSR International Pty Ltd, Doncaster, Australia).

We avoided making a priori hypotheses and conclusions, in keeping with a grounded theory approach [21]. The initial thematic indices were developed, with the addition of emerging thematic categories according to interpretation of the content of the data. The analysis proceeded through three stages, consisting of open, axial, and selective coding, with constant comparison taking place throughout each phase [21]. Each stage provided categories that could be used to explore the themes of the data.

## Results

A total of 27 students consented to attend interviews in June 2016, after they had completed their end of year examinations.

Following the thematic analysis approach stated above, the participant interviews specified five core themes: (1) Aims of the incident reporting system; (2) internalized cognition of the system; (3) the impact of the reporting system; (4) threshold for reporting; (5) feedback on the systems operation. Quotes from participants have been included to evaluate the credibility of the overall analysis [20] and are presented below for each theme. Participant numbers are presented as P1–27.

### Perceptions of purpose of system

The open coding produced two primary purposes of the reporting system which students thought the system utilized.

#### Patient and student safety

Students considered that the aim of the incident reporting system was to support patient safety. The central purpose of the system was thought to be: ‘*First of all to maintain the safety of the patients’* (P9).

Other than patient safety, students believed that the reporting system was also there to protect students themselves, which in turn was beneficial in ensuring patient safety: *‘It’s kind of protecting your students as well. Protecting ourselves and then if it protects us, it helps future patients we work with*’ (P16).

#### Control of behaviour

Medical students also believed that the purpose of the incident reporting system was centred around the ‘control’ of student behaviour. The system was training students to change their behaviours by ‘shaping’ or ‘moulding’ them to a certain type of person in order to ‘quality control each other’. In that way, students would be reported ‘in order to control their actions’: *‘If someone deviated – someone not performing at that level, you can tell them you’re not doing well enough*’ (P4).

Five students also described the safeguarding purposes of the system: *‘Yeah, if you don’t share it, nobody knows … So somebody knows and you can work around it’* (P24). And: *‘For that kind of situation, then I think it’s good, because you need to know whether somebody has a mental health issue or they are not coping and that kind of system does work because you notify that at this specific time, that someone is battling or having mental health issues. Maybe later on, they might not have it. So that was a significant event maybe’ (P7).*

### Internalized cognition of the system

Acknowledging students’ beliefs of why the incident reporting exists has created views on how students have internalized their feelings and emotions about the reporting system. They perceive the system as a ‘very serious thing’ that has the ability to affect the future of medical students, hence causing ‘unnecessary stress’. The view that being reported means they have done something wrong aligns with the aims of controlling student behaviour by stating their current behaviour is not good enough and needs to change: *‘Some students might be too frustrated to get a SERF, even if it’s a constructive one because it’s like there’s something on your record which is, which indicated that you did something not so good in the past …*’ (P22).

Due to this perception of the system, reporting has reinforced the idea that incident reporting was created to blame students rather than for learning from their errors; something which has been predominantly used in the past [19]. This led students to believe that the system was designed to punish them rather than to help them: ‘*It is penalizing them for maybe something they have said’* (P2). This issue of blame led five students to comment regarding its place in their portfolio: *‘I am worried a report is on my record forever whatever the SERF’ *(P3). This caused students to feel vulnerable, internalizing their feelings of being scared and frightened of getting a SERF: ‘*We don’t want to be threatened with SERFs, even if we think it might be to help us’* (P14). And: *‘I think it is more about negative than positive reporting and I don’t want to get my name mingled in with SERF’* (P2).

### Impact of the reporting system

The internal feelings and emotions of students are associated with the impact the reporting system has on students. Feeling frightened and seeing the reporting system as a threat has increased caution amongst students regarding the types of behaviour displayed in their surroundings, although intimate environments are an exception. This ultimately controlled their behaviour: *‘I’m still watching what I’m saying here because I know that anything I say here could be used against me. I watch what I say when I go out, I watch what I say when I go nearly everywhere except in special places (laughs from all)’ (P26).*

Interestingly, this quote was met by laughs from the rest of the focus group, most likely because they empathized with the student’s comment. However, not all students felt the impact of the reporting system this way. Instead, some students played down the significance of the way the system impacted on them: *‘I don’t really care about SERF; I’m not too bothered – that’s what I think’* (P2).

### Threshold for reporting

The incident reporting system takes in significant events, but students believe that minor events should also be reported: *‘Constructive things can be small things; it doesn’t have to be a significant event for it to be reported*’ (P1).

The response to ‘minor’ events was negatively internalized by students as they ‘*got a bit frightened because they thought it was a very minor incident that happened*’ (P3). Furthermore, students who were reported and perceived their event as minor, felt that these minor events did not benefit the incident reporting as a whole, as one student states that: *‘if it’s a minor event, that’s put on there – sort of wasting time and wasting the potential of the SERF in the future because people are associating it with something. I want to say dumb but that’s been put on there previously. So it is sort of devaluing the whole process’* (P10).

### Feedback on the systems operation

In their feedback, students mentioned some ineffective aspects of the incident reporting system.

Subjectivity: A core theme (11 reports) was students perceiving that the reporting system was not objective enough, drawing the conclusion that the system was open to interpretation and biased, varying differently from teacher to teacher: *‘Cases of it the first year where two students did similar things, one of them got a negative and the other didn’t’* (P5).

This led to comments regarding alternative routes that could be used instead of SERF reporting: *‘I think there is a small problem going on with my class mate, I wouldn’t – SERFing them, for me, would not solve the problem; it’s not getting to the root of it’* (P12) and *‘We can sort it out one on one, having one on one conversations with each other instead of reporting’ *(P8).

However, students did think that the incident reporting system had some benefits to their wider learning: *‘This is part of our gradual learning process for us students to gradually become a doctor and it’s always good to have a third party – a sort of unbiased perspective to see how the situation might be’* (P6).

At the selective level of coding, there were three key overriding findings: lack of error awareness and error wisdom as underpinned by key theoretical constructs [11, 19], students supporting the principle of safety and perceptions of a blame culture.

## Discussion

The aims of this paper were to describe a medical school incident reporting system and identify the participating students perception of safety. The presented data appear to show appropriate use and engagement with the system (Table 1). However, the analysis of interview data seems to suggest difficulties with the system. Students perceived that the primary goal of reporting was not only to safeguard, but also to control behaviours. Paradoxically, students still felt the system was advantageous for all involved. Students also pinpointed the safeguarding aspect of reporting as effective in protecting them, but as patient contact was limited in the early phases of the course this was not well discussed.

Multiple comments (13 students) were made regarding students being scared of being reported, perceiving the system as a threat, hence causing a misappropriation of a blame culture [11]. This culture of fear will impact engagement with the system [8]. Students did not state that they had gained understanding of why errors occur, nor did they acquire personal non-technical skills in error awareness, even though such skills have previously been reported after introduction of other safety teaching packages [22] and error feedback systems [23].

Students believed that incident reporting was subjective and open to interpretation with reports differing between students. Furthermore, some students thought reporting made it worse and was not a solution that did anything other than record an event. Therefore, alternative solutions were identified to rectify incidents. Similar to Mitchell et al. [15], the reporting system included triaging and taking effective action, which students felt was beneficial in safeguarding compared with the professionalism aspect of reporting, but they were concerned about the subjectivity of these judgements, further seeing this as being punitive and ‘not fair’.

This is first paper to the authors’ knowledge to describe student participation in an incident reporting system within medical school. Whilst we maintain the rationale of such a process of learning through doing with a key aspect of safety culture within healthcare, the findings were unexpected. This particularly raises questions as to whether the underpinning hypothesis that early exposure to such a system can support acquisition of safety behaviour is correct. As such, several amendments have been made to the system. Firstly, the triaging is no longer the responsibility of those reporting the incident and such judgements are made by the officers receiving the reports, with judgement quality checked on a regular basis. This was to address the apparent issues about the fairness of such judgements and the perception that this was not just about speed of response but the seriousness of the response needed. Secondly, student training has been intensified with two sessions of 30 min during induction and two similar follow-up sessions in the first term, with anonymous presentation of data and discussion of real-life examples. Similarly, faculty training has been increased, focussing on the underpinning theory and the purpose of the system, not just to achieve safety but also to foster students learning about safety. This consists of termly sessions lasting two hours, with all the staff attending at least once a year and real-life examples discussed, as well as the results of this study.

The key limitation of this study is the context in which it was carried out. This a new medical curriculum and as such the students do not have senior peers to draw on for support. Additionally, all students are international from over 40 countries and as they are early in their training, clinical placements where such a system will be key are currently limited.

Therefore, future research should aim to investigate whether student views change with future learning within the curriculum and in particular considering the changes in how the system is run and increasing clinical placements for students. In addition, it may also be necessary to identify alternative methods that could possibly challenge the development of a construct of blame within reporting; methods to achieve this based on previous reports are being designed [23].

## Conclusions

This first report of a medical school system of incident reporting, open for all to use, has been instituted and run to support dissemination and replication in other institutions. It was found that students accept the safeguarding aspect of reporting. However, they did not demonstrate development of relevant non-technical skills and were concerned about a blame culture, which is at odds with the goals of the system. This led to key changes in both the triaging of reports and training of staff and students. Future research is needed to observe if student perceptions change with future learning within the curriculum and as a result of the amendments to the system. In addition, research needs to identify alternative methods that could challenge the development of a construct of blame within reporting.
